# Clinical Audit and Re-audit on Valproate Monitoring of Inpatients at DHQ Hospital, Faisalabad

**DOI:** 10.1192/bjo.2025.10577

**Published:** 2025-06-20

**Authors:** Duaa Fatima, Imtiaz Ahmad Dogar, Palvisha Sajid, Sammar Fatima, Sinha Tahir

**Affiliations:** Faisalabad Medical University, Faisalabad, Pakistan

## Abstract

**Aims:** The aim of this audit was to ascertain the number of patients prescribed valproate who underwent pre-prescription evaluation and ongoing treatment monitoring was in alignment with NICE guidelines.

**Methods:** The study, conducted in early 2024 at a tertiary care hospital in Faisalabad, involved 18 bipolar affective disorder patients (12 male, 6 female) prescribed valproate. A baseline audit was followed by an intervention – a faculty-led presentation on monitoring guidelines – and a re-audit three months later. Data from patient files (1–30 April) was reviewed using an audit tool to assess compliance with NICE standards. Initial findings revealed unsatisfactory monitoring. Results were discussed in a departmental meeting, leading to targeted training for residents. The re-audit showed significant improvement in monitoring practices, demonstrating the effectiveness of the intervention.

**Results:** The initial audit revealed that only 3 out of 18 patients (16%) had baseline weight measurements, and 6 (33%) had full blood count and liver function tests during treatment. A re-audit conducted months later reviewed 25 patient charts, showing marked improvement: 20 patients (80%) had baseline weight checked, and 18 (72%) underwent recommended blood tests during treatment. These findings indicate a positive staff response and significant progress in adhering to monitoring guidelines for patients on valproate.

**Conclusion:** This audit underscores the critical need for comprehensive monitoring in patients prescribed valproate. The drug is associated with increased risks of insulin resistance, obesity, Type II diabetes, and cardiovascular complications. Valproate also impacts haematopoietic homeostasis, inhibiting erythroid differentiation and activating the myelo-monocytic pathway, leading to thrombocytopenia manifesting as prolonged bleeding, platelet abnormalities, or petechial bleeding. Hepatotoxicity ranges from mild ALT elevation to severe liver injury, including jaundice, hepatic dysfunction, coma, or death. Rigorous monitoring of these parameters is vital for mitigating risks, enhancing patient safety, and improving quality of care through effective primary prevention strategies.

In conclusion, this audit highlights the critical importance of adhering to NICE guidelines for valproate monitoring to ensure patient safety and minimize potential adverse effects. The significant improvement observed in the re-audit demonstrates the effectiveness of targeted educational interventions, underscoring the need for ongoing staff training and regular audits to maintain compliance and enhance patient care.

